# Integrated Analysis of Hepatic miRNA and mRNA Expression Profiles in the Spontaneous Reversal Process of Liver Fibrosis

**DOI:** 10.3389/fgene.2021.706341

**Published:** 2021-07-22

**Authors:** Yang Tai, Chong Zhao, Tian Lan, Linhao Zhang, Yang Xiao, Huan Tong, Rui Liu, Chengwei Tang, Jinhang Gao

**Affiliations:** ^1^Laboratory of Gastroenterology and Hepatology, State Key Laboratory of Biotherapy, West China Hospital, Sichuan University, Chengdu, China; ^2^Department of Gastroenterology, West China Hospital, Sichuan University, Chengdu, China

**Keywords:** miRNA, mRNA, liver fibrosis, spontaneous reversal, extracellular matrix, lysyl oxidase

## Abstract

Liver fibrosis results from the imbalance between extracellular matrix (ECM) production and degradation, which is a common pathological consequence of various chronic liver diseases. Although many miRNAs have been reported in liver fibrosis progression, miRNA-mRNA interactions in its reversal process remain to be elucidated. In the current study, we performed an integrated analysis of miRNA and mRNA expression profiles in the mouse model with the spontaneous reversal potency of liver fibrosis. A total of 102 miRNA and 2,845 mRNAs showed significant differential expression in reversal mice compared to fibrotic mice. Moreover, 3,769 putative negatively correlated miRNA-mRNA pairs were revealed to be potentially implicated in the biological function regulation of small molecule metabolism and ECM organization. By integrating miRNA-mRNA regulatory networks, mmu-miR-1843a-5p, mmu-miR-193a-5p, mmu-miR-194-2-3p, and mmu-miR-30c-2-3p were identified as lysyl oxidases-specific miRNAs that were correlated with fibrosis reversal. Our results provided potential candidate targets for the treatment of liver fibrosis.

## Introduction

Liver fibrosis, characterized by the excessive deposition of extracellular matrix (ECM), is a common pathological consequence of various chronic liver diseases, including viral hepatitis, alcoholic and metabolic associated liver diseases (Tsochatzis et al., 2014). Essentially, liver fibrosis is an abnormal wound-healing process response to chronic liver injuries due to various etiological factors (Kisseleva and Brenner, 2021). It was ever believed that liver fibrosis was not reversible. Nevertheless, several studies with sequential liver biopsies have already shown that hepatic fibrosis could be reversed in patients with hepatitis B, hepatitis C, non-alcoholic steatohepatitis (NASH), autoimmune hepatitis and secondary biliary fibrosis after the underlying causes of liver injury are removed (Hammel et al., 2001; Kweon et al., 2001; Arthur, 2002; Czaja and Carpenter, 2004; Dixon et al., 2004). However, the biological molecular mechanism regarding the spontaneous reversal of liver fibrosis is unclear.

MiRNAs are a class of evolutionarily conserved small (∼23 nucleotides in length), single-stranded non-coding RNA generated from the endogenous transcripts following a series of cleavages and modifications (Bartel, 2009). Mature miRNAs act as post-transcriptional regulators via the degradation or translational repression of the target mRNAs by binding to the complementary sequences. It has been estimated that over 30% of human genes are regulated by miRNAs, and one single miRNA may modulate hundreds of target mRNAs (Lewis et al., 2005). Recently, a competitive endogenous RNA hypothesis has been proposed that long non-coding RNAs and circular RNAs may serve as “miRNA sponges” by preferentially occupying the miRNA response elements to regulate mRNAs expression (Thomson and Dinger, 2016), highlighting the central position of miRNAs in cellular function. Accumulated evidence has demonstrated that the dysregulated miRNAs play a crucial role in the development of liver fibrosis by interfering with genes expression and downstream signaling pathways (Wang et al., 2021). Serous miR-122 may serve as a biomarker of inflammation and fibrotic severity in patients with alcoholic liver disease (Satishchandran et al., 2018) and hepatitis C (Bihrer et al., 2011; Trebicka et al., 2013). miR-155 and miR-378 exert pro-inflammatory effects by positively regulating the NF-κB signaling pathway in alcoholic and NASH induced fibrosis, respectively (Bala et al., 2016; Zhang et al., 2019). Thus far, little is known about miRNAs expression profile and their potential functions in the spontaneous reversal of liver fibrosis.

Ongoing liver fibrosis associated with angiogenesis may progress into irreversible cirrhosis with life-threatening complications even inactivation of primary diseases (Tsuchida et al., 2018). Therefore, it is very important to understand the biological molecular mechanism especial in expression of miRNAs and their target mRNAs related to fibrosis regression during the spontaneous reversal of liver fibrosis, by which it would be benefit for prevention of liver cirrhosis on the basis of etiological treatment.

In the present study, we analyzed the expression profiles of miRNAs and mRNAs in the mouse model with the spontaneous reversal potency of liver fibrosis on cessation of liver injuries after given sufficient time. Furthermore, the candidate miRNA-mRNA interactive networks were also defined preliminarily.

## Materials and Methods

### Animal Model and Sample Collection

*C57BL/6* mice (male, 8-week-old), weighing 20–22 g, were obtained from the Experimental Animal Center of Sichuan University (Chengdu, China). All animals were kept under the 12-h light/dark cycles with free access to food and water. The procedure for animal experiments was approved by the Animal Use and Care Committee of West China Hospital, Sichuan University (No. 2017005A).

Liver fibrosis of mice was induced by intraperitoneal injection of thioacetamide (TAA, 200 mg/kg, Sigma-Aldrich, United States) every 3 days for 8 weeks. Control mice received the same volume of normal saline. Control and TAA-fibrotic mice were sacrificed under anesthesia 72 h after the final TAA injection. The liver fibrosis reversal model of mice was firstly established with the same 8-week TAA injection as above. Then, the animals underwent spontaneous recovery for additional 8 weeks. Liver tissues were fixed with 4% paraformaldehyde (PFA) for histological and immunohistochemical examinations, or immediately snap-frozen in liquid nitrogen and stored at −80°C for further gene and protein analysis.

### Histological Examination

Liver samples fixed with 4% PFA were sectioned (5 μm thick) after embedded in paraffin and stained with hematoxylin and eosin (H&E) and Sirius Red. Five images per section (at × 100 magnification) from each mouse were selected and assessed by two experienced pathologists independently blinded to the experimental groups. The severity of liver fibrosis was graded according to the Ishak scoring system (Ishak et al., 1995).

### Immunohistochemistry Staining

Paraffin-embedded liver sections (3 μm   thick) were deparaffinized in xylene and rehydrated with graded ethanol dilutions. Antigen retrieval was performed at high temperature under high pressure in sodium citrate buffer (10 mM, pH = 6.0) for 20 min. After blocking with H_2_O_2_ and 10% goat serum, the sections were incubated with anti-collagen I (1:200, Abcam, United Kingdom) or anti-α-smooth muscle actin (α-SMA, 1:200, Abcam) overnight at 4°C followed by incubation with biotin-streptavidin-horseradish peroxidase (HRP) detection system (ZSGB-BIO, Beijing, China) at room temperature. Finally, the sections were stained with a solution of 3, 3’-diaminobenzidine (DAB, ZSGB-BIO) and counterstained with hematoxylin.

### Hepatic Hydroxyproline Measurement

Hepatic hydroxyproline content, a major component of collagen, was measured in 400–500 mg liver samples using the Hydroxyproline Assay Kit (Nanjing Jiancheng Bioengineering Institute, Nanjing, China) according to the manufacturer’s instructions and expressed as microgram per gram tissue (μg/g liver).

### Western Blot Analysis

Whole proteins from liver tissues were extracted using a protein extraction kit (Nanjing KeyGen Biotech Co., Ltd., Nanjing, China). 50 μg (7.2 μg/μL, 7 μL) of proteins for each sample were resolved by 10% SDS-PAGE, transferred to PVDF membrane (Merck Millipore, United States), and blocked with 5% non-fat dry milk before incubated with anti-collagen I (1:1,000, Abcam), anti-α-SMA (1:4,000, Abcam) or anti-HSC70 (1:1,000, Santa Cruz Biotechnology, United States) overnight at 4°C. Then, the blots were washed and incubated with HRP-conjugated secondary antibodies (1:20,000, ZSGB-BIO) at room temperature for 2 h. Protein bands were visualized by chemiluminescence using Western Blotting Luminol Reagent (Merck Millipore), and densitometric analyses were made using Quantity One software (v4.6.2). Protein levels were normalized against HSC70 and were shown as fold changes relative to the control group.

### RNA Extraction and Quality Control

To obtain miRNA and mRNA expression profiles, total RNA was extracted from liver samples using TRIzol reagent (Thermo Fisher Scientific, United States). Next, the RNA samples were qualified and quantified as follows: RNA degradation and contamination were monitored on 1% agarose gels; RNA purity and concentration were then examined using the NanoPhotometer spectrophotometer (Implen, Germany); RNA integrity and concentration were finally assessed and quantified using the RNA Nano 6,000 Assay Kit of the Bioanalyzer 2,100 system (Agilent Technologies, United States). The data on the quality of RNA were provided in Supplementary Figure 1.

### Small RNA Sequencing (Small RNA-Seq) and Data Analysis

Small RNA libraries were constructed using the NEBNext Multiplex Small RNA Library Prep Kit for Illumina (NEB, United States), following the manufacturer’s instructions. Then, the purified libraries were quantified by Qubit 2.0 Fluorometer (Thermo Fisher Scientific), and validated by Agilent 2,100 bioanalyzer (Agilent Technologies). After cluster generation, the final library preparations were submitted for small RNA-seq on the Illumina-HiSeq 2500 platform (Illumina, United States) and 50 bp single-end reads were generated. Raw data were processed through custom perl and python scripts, and were then filtered by Q20, Q30, and GC contents. The acquired clean data were mapped to the mouse reference genome (mm10) and aligned against miRbase (Release 22.1) by Bowtie (v1.3.0) (Langmead et al., 2009) to identify mature miRNAs. For each sample, the miRNA expression profiles were normalized by transcript per million (TPM), as previously reported (Zhou et al., 2010). Differentially expressed miRNAs (DEMs) were analyzed using DESeq2 (v1.16.1), with a significance threshold of false discovery rate (FDR) < 0.05 and |fold change| ≥ 2.

### mRNA Sequencing (RNA-Seq) and Data Analysis

For RNA-seq, 1 μg of total RNA per sample was used for library preparation. The NEBNext Ultra RNA Library Prep Kit for Illumina (NEB) was used for library construction, according to the manufacturer’s protocols. After purification and quantification, the libraries were subjected to 150bp paired-end sequencing on the Illumina NovaSeq platform (Illumina). As described above, quality-filtered reads were then mapped to the mouse reference genome (mm10) using HISAT2 (v2.2.1) (Kim et al., 2019). Read counts for each gene were obtained with featureCounts (v1.5.0-p3) and were standardized as fragments per kilobase million (FPKM). DESeq2 (v1.16.1) was used to analyze differentially expressed genes (DEGs) with a filter criterion of FDR < 0.05 and |fold change| ≥ 2. Heatmaps and volcano plots were generated to visualize gene expression patterns using TB (Toolbox for Biologists) tools (v1.082) (Chen C. et al., 2020) and GraphPad Prism 8.4.0 (GraphPad, United States), respectively. Small RNA-seq and RNA-seq were performed by Novogene (Beijing, China).

### miRNA-mRNA Integrated Analysis

The target genes of DEMs were predicted by an online tool miRWalk^1^ (Sticht et al., 2018), which collates data from multiple prediction programs (TargetScan, miRDB and miRTarBase). The intersections between the predicted target genes and DEGs were screened out for further Pearson correlation analysis. Since miRNA mainly inhibits the translational activity of its target mRNA, negatively paired miRNA-mRNA correlations were selected, according to the screening criteria of a *p-*value < 0.05 and a Pearson correlation coefficient (PCC) ≥ 0.8. DEGs involved in the negative miRNA-mRNA pairs were subjected to subsequent analysis. Then, the miRNA-mRNA interaction networks were constructed and visualized using Cytoscape software (v3.8.2).

### Functional Enrichment Analysis

To explore the functional roles of these significant DEGs, GO enrichment and KEGG pathway analysis were performed using Metascape^2^ (Zhou et al., 2019). The GO terms and KEGG pathways with FDR less than 0.05 were considered significantly enriched, and visualized by online bioinformatics platforms.^3^ Additionally, the enriched Reactome pathway interaction networks were constructed using Cytoscape plug-in ClueGO (v2.5.7).

### Protein-Protein Interaction (PPI) Network

The differentially expressed target genes were mapped to the STRING database^4^ (Szklarczyk et al., 2019) to screen PPI, and results were visualized by Cytoscape software as a network structure.

### Quantitative Real-Time PCR (qRT-PCR)

Total RNA was extracted from the frozen liver tissues using an RNA Isolation Kit (Foregene, Chengdu, China), according to the manufacturer’s instructions. Then, equal amounts of 1 μg RNA were reverse-transcribed into cDNA using RevertAid^TM^ First-Strand cDNA Synthesis Kit (Thermo Fisher Scientific) or miRcute Plus miRNA First-Strand cDNA Synthesis Kit (Tiangen, Beijing, China), respectively. qRT-PCR was performed in triplicate using SYBR Green qPCR Master Mix (Bimake, United States) on the CFX96 Real-Time PCR Detection System (Bio-Rad). Expression of miRNA and mRNA were normalized to U6 snRNA or *Gapdh*, and were shown as fold changes relative to the control group. Primer sequences were listed in Supplementary Table 1.

### Statistical Analysis

All data were expressed as mean ± standard deviation and analyzed by SPSS 19.0 software (SPSS, United States). The Student’s *t*-test was performed for comparisons between two groups, and one-way ANOVA (Students-Newman-Keuls test) was used for comparisons of multiple groups. A *p-*value < 0.05 was considered statistically significant.

## Results

### Model Establishment of the Spontaneous Reversed Liver Fibrosis

**FIGURE 1:**
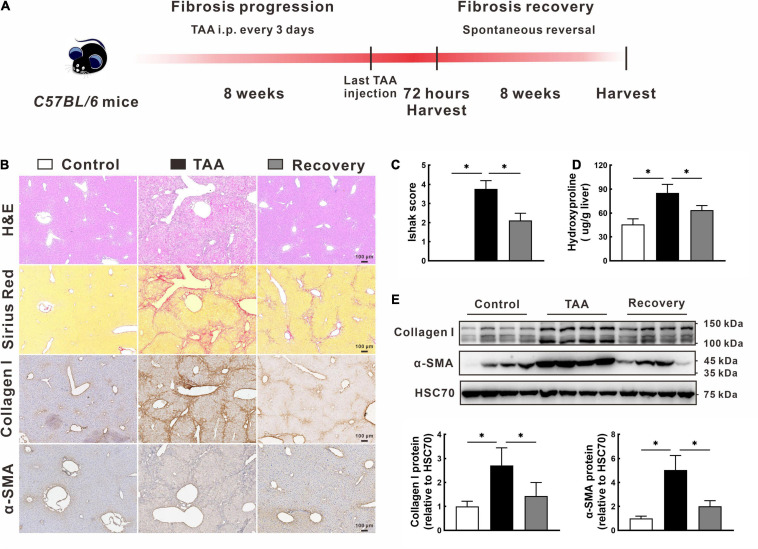
Model establishment of the spontaneous reversed liver fibrosis. **(A)** Flow diagram of the animal treatment procedure for establishing the spontaneous reversed liver fibrosis model. **(B–D)** Representative liver histology of H&E and Sirius Red staining, immunohistochemistry of collagen I and α-SMA **(B)**, and quantification of hepatic fibrosis as measured by the Ishak score **(C)** and hepatic hydroxyproline content **(D)**. **(E)** Expression of collagen I and α-SMA were determined by Western blot. Data were presented as mean ± SD. *n* = 6/group, *p < 0.05. α-SMA, α-smooth muscle actin; GAPDH, glyceraldehyde-3-phosphate dehydrogenase; H&E, hematoxylin and eosin; TAA, thioacetamide.

Following the animal treatment procedure shown in Figure 1A, we successfully established a mouse model of spontaneous reversed liver fibrosis. At the end of 8-week modeling, liver tissues in the TAA group showed a typical advanced fibrosis appearance with disordered hepatic lobular structure and continuous fibrotic septa (Figure 1B). However, the pathological characteristics of liver fibrogenesis and liver injury were remarkably regressed after TAA withdrawal for additional 8 weeks (Figure 1B). In support of this, Ishak score and hepatic hydroxyproline content in the recovery group were 43.6% and 25.2% lower than those in the TAA group, respectively, *p* < 0.05 (Figures 1C,D). Additionally, the protein levels of collagen I and α-SMA were also significantly decreased in the recovery group compared with the TAA group (Figures 1B,E).

### Identification of DEMs and DEGs

**FIGURE 2:**
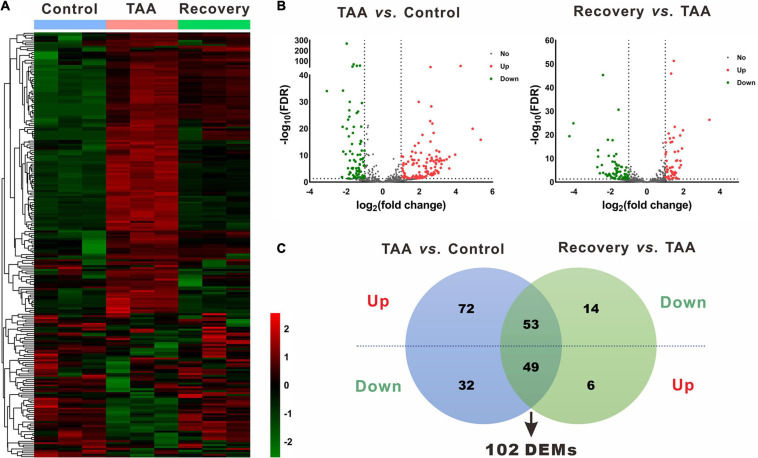
Identification of differentially expressed miRNAs (DEMs). **(A)** Heatmap of miRNAs via hierarchical cluster analysis. Colors from green to red represented the miRNA expression abundance from poor to rich. **(B)** Volcano plots of miRNAs between TAA and Control, and Recovery and TAA, respectively. Red dots represented up-regulated miRNAs, green dots represented down-regulated miRNAs, and gray dots represented miRNAs with no significant difference. **(C)** Venn diagram of DEMs between TAA-fibrotic mice and reversal mice. DEMs, differentially expressed miRNAs; FDR, false discovery rate; TAA, thioacetamide.

**FIGURE 3:**
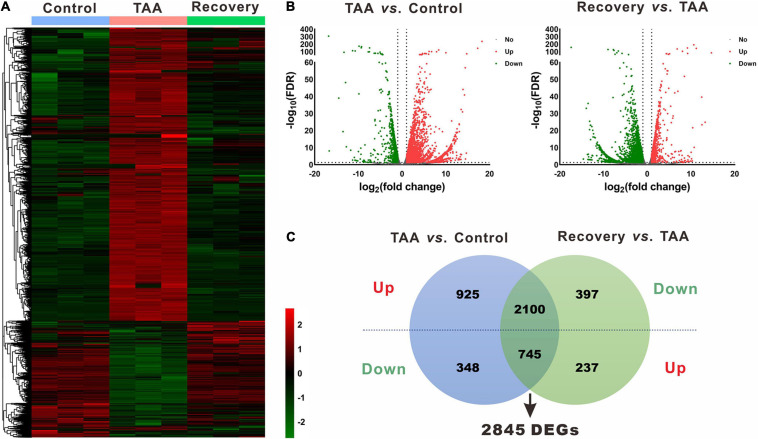
Identification of differentially expressed genes (DEGs). **(A)** Heatmap of mRNA via hierarchical cluster analysis. Colors from green to red represented the mRNA expression abundance from poor to rich. **(B)** Volcano plots of mRNAs between TAA and Control, and Recovery and TAA, respectively. Red dots represented up-regulated mRNAs, green dots represented down-regulated mRNAs, and gray dots represented mRNAs with no significant difference. **(C)** Venn diagram of DEGs between TAA-fibrotic mice and reversal mice. DEGs, differentially expressed genes; FDR, false discovery rate; TAA, thioacetamide.

In the miRNA and mRNA expression profiling by deep sequencing, 9 mice were selected from the control (*n* = 3), TAA (*n* = 3) and recovery group (*n* = 3). DEMs and DEGs were visualized in heatmaps (Figures 2A, 3A) and volcano plots (Figures 2B, 3B). Information on the top 20 DEMs and top 20 DEGs in detail was listed in Tables 1, 2, respectively. According to the screening criteria of FDR < 0.05 and |fold change| ≥ 2, 206 miRNAs were differentially expressed in the TAA group compared with the control group, among which 102 DEMs were reversed in the recovery group (Supplementary Table 2). Furthermore, 53 miRNAs were up-regulated in TAA-fibrotic mice while their expression was suppressed in mice with reversed liver fibrosis. There were 49 miRNAs down-regulated in TAA-fibrotic mice while rescued in mice with reversed liver fibrosis (Figure 2C). Among these DEMs, mmu-miR-592-5p was overexpressed with the highest fold change of 10.67. In contrast, mmu-miR-344d-3p was downregulated with the highest fold change of 18.96.

**TABLE 1:** The top 20 differentially expressed miRNAs in liver fibrosis reversal.

**Up-regulated miRNAs**	**Fold change**	**FDR**	**Down-regulated miRNAs**	**Fold change**	**FDR**
mmu-miR-592-5p	10.67	4.45E−27	mmu-miR-344d-3p	−18.96	3.78E−20
mmu-miR-29c-3p	3.92	1.17E−22	mmu-miR-218-5p	−16.31	1.37E−25
mmu-miR-455-3p	3.77	6.26E−15	mmu-miR-296-3p	−6.58	4.55E−07
mmu-miR-1843a-3p	3.62	7.16E−08	mmu-miR-190b-5p	−6.40	3.23E−14
mmu-miR-193a-5p	3.55	9.97E−10	mmu-miR-582-3p	−6.37	1.49E−11
mmu-miR-365-3p	3.52	1.88E−20	mmu-miR-3079-5p	−5.99	1.86E−04
mmu-miR-376b-3p	3.43	7.79E−07	mmu-miR-582-5p	−5.55	6.27E−08
mmu-miR-7240-5p	3.27	3.63E−02	mmu-miR-34a-5p	−5.32	5.09E−46
mmu-miR-122-5p	3.26	9.04E−14	mmu-miR-298-5p	−5.28	1.17E−04
mmu-miR-122b-3p	3.26	9.04E−14	mmu-miR-147-3p	−4.79	4.94E−04
mmu-miR-202-5p	3.23	3.76E−02	mmu-miR-135a-5p	−4.51	2.83E−03
mmu-miR-7219-3p	3.14	6.48E−10	mmu-miR-34c-5p	−4.41	1.19E−18
mmu-miR-215-3p	3.03	1.36E−02	mmu-miR-497b	−4.37	9.64E−04
mmu-miR-1948-5p	3.02	9.97E−10	mmu-miR-200b-5p	−4.28	7.55E−12
mmu-miR-193a-3p	2.99	1.23E−04	mmu-miR-18a-5p	−4.18	3.36E−04
mmu-miR-455-5p	2.92	6.45E−05	mmu-miR-3081-3p	−4.05	1.49E−03
mmu-miR-194-2-3p	2.87	1.67E−13	mmu-miR-467a-3p	−3.87	6.75E−03
mmu-miR-203-3p	2.86	4.20E−24	mmu-miR-467d-3p	−3.87	6.75E−03
mmu-miR-5615-5p	2.85	8.28E−04	mmu-miR-873a-5p	−3.76	1.79E−02
mmu-miR-5615-3p	2.81	1.02E−03	mmu-miR-200a-5p	−3.70	2.12E−09

*FDR, false discovery rate.*

**TABLE 2:** The top 20 differentially expressed mRNAs in liver fibrosis reversal.

**Up-regulated mRNAs**	**Fold change**	**FDR**	**Down-regulated mRNAs**	**Fold change**	**FDR**
*Mup19*	2.60E+04	2.20E−86	*Cyp3a16*	−1.73E+05	8.78E−158
*Mup15*	5.79E+03	3.62E−24	*Rdh16f1*	−2.49E+04	4.83E−04
*Mup8*	5.57E+03	2.34E−40	*Sprr1a*	−1.57E+04	8.85E−34
*Mup1*	2.36E+03	2.33E−144	*Acta1*	−1.31E+04	9.55E−07
*Meig1*	1.69E+03	6.86E−11	*Myh2*	−1.21E+04	8.31E−04
*Elovl3*	1.60E+03	6.03E−193	*Cfap44*	−1.18E+04	1.19E−36
*Mup17*	1.35E+03	2.31E−22	*Actn2*	−1.09E+04	2.21E−05
*Cyp11b1*	1.25E+03	5.87E−03	*Ckmt2*	−1.08E+04	9.35E−04
*Sult3a1*	1.05E+03	5.11E−08	*Usp9y*	−1.05E+04	9.56E−04
*Gabra5*	9.88E+02	9.15E−03	*Myl1*	−9.72E+03	1.03E−03
*Cyp11a1*	9.66E+02	8.19E−03	*Serpinb9f*	−9.36E+03	2.94E−21
*Cfd*	7.90E+02	2.34E−05	*Acsm4*	−9.21E+03	3.43E−26
*Rspo2*	7.39E+02	1.19E−02	*Pvalb*	−8.29E+03	1.19E−03
*Tmem132d*	6.35E+02	1.47E−02	*S100g*	−8.23E+03	8.44E−24
*Cdh9*	6.24E+02	1.55E−03	*Npy*	−7.99E+03	1.99E−15
*Mup7*	5.98E+02	2.09E−77	*Adamts16*	−7.92E+03	6.89E−24
*Slc1a2*	5.79E+02	1.78E−150	*Myh4*	−7.79E+03	1.28E−03
*Cntnap5a*	5.19E+02	1.91E−02	*Mybpc1*	−7.17E+03	5.91E−05
*St18*	3.69E+02	4.99E−03	*Tmc5*	−6.52E+03	4.43E−23
*Mup16*	3.61E+02	1.61E−52	*Arhgap26*	−6.47E+03	1.57E−03

*FDR, false discovery rate.*

Using the same criteria as for miRNAs, a total of 4,118 DEGs were identified in liver fibrosis. Notably, 2,100 out of 3,025 up-regulated mRNA transcripts upon TAA stimulation were suppressed on cessation of TAA exposure, while 745 out of 1,093 down-regulated mRNA transcripts were rescued simultaneously followed by the withdrawal of repeated liver damages (Figure 3C and Supplementary Table 3). Within the identified 2,845 DEGs, *Cyp3a16*, *Rdh16f1*, *Sprr1a*, *Acta1*, *Myh2*, etc. were decreased, while *Mup19*, *Mup15*, *Mup8*, *Mup1*, *Meig1*, etc. were increased. Overall, miRNA and mRNA with similar expression patterns in control and reversal mice and opposite to fibrotic mice may potentially relate to the progression and regression of liver fibrosis.

### Target Gene Identification and Functional Enrichment

**FIGURE 4:**
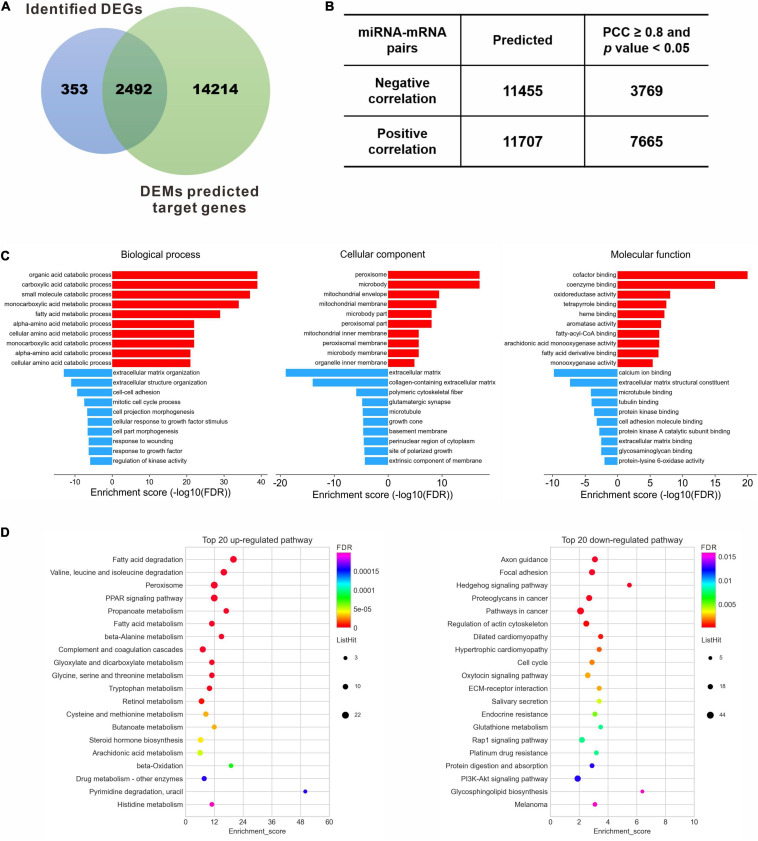
Target gene identification and functional enrichment. **(A)** Venn diagram between identified DEGs and predicted target genes of DEMs. **(B)** Pearson correlation analysis to identify the negative correlating miRNA-mRNA pairs. **(C)** Top 10 enriched GO terms of the corresponding biological process, cellular component and molecular function for the up- and down-regulated DEGs, respectively. **(D)** Top 20 enriched KEGG pathways for the up- and down-regulated DEGs, respectively. DEGs, differentially expressed genes; DEMs, differentially expressed miRNAs; FDR, false discovery rate; PCC, Pearson correlation coefficient.

One hundred and two DEMs and 2,845 DEGs identified in the previous steps were considered for the miRNA-mRNA integrated analysis. A total of 16,706 genes were consistently predicted as potential targets of 102 DEMs using an online bioinformatics database miRWalk. Then a Venn diagram showed that 2,492 overlapped genes were obtained between the predicted target genes and the identified DEGs (Figure 4A), which were selected for further correlation analysis. According to the standard of absolute PCC ≥ 0.8, we finally identified 11,434 differentially expressed miRNA-mRNA pairs, including 3,769 negative correlating pairs and 7,665 positive correlating pairs (Figure 4B). Since miRNAs generally suppress the expression of target mRNAs, those pairs where the miRNA and mRNA expression levels changed in the same direction were filtered out. In the case of 3,769 negative miRNA-mRNA interaction pairs, 2,168 pairs that composed 23 miRNA and 1,056 mRNA were up-regulated miRNAs *vs.* down-regulated mRNAs, while 1,601 pairs composed 53 miRNA and 434 mRNA were down-regulated miRNAs *vs.* up-regulated mRNAs (Supplementary Table 4).

To understand the biological function of these dysregulated target genes, GO enrichment and KEGG pathway analysis were performed for the 1,490 DEGs. GO analysis revealed that the up-regulated DEGs were enriched in the metabolic process of lipid and amino acids, peroxisome and membrane components, and protein binding and enzyme activity, while the down-regulated DEGs were enriched in terms related to ECM organization, such as cell-cell adhesion, response to growth factor, and polymeric cytoskeletal fiber (Supplementary Table 5). Ten most significantly enriched GO terms, involving biological processes, cellular component and molecular function, were shown in Figure 4C. The correlating transcripts were also analyzed for enrichment in KEGG pathway categories (Supplementary Table 6), and the top 20 KEGG pathways were identified for the up- or down-regulated DEGs (Figure 4D). Consistent with GO analysis, lipid and amino acid metabolism were significantly enriched by KEGG pathway analysis for the up-regulated genes (Figure 4D, left panel). In contrast, the top 20 down-regulated genes were mainly enriched in regulation of actin cytoskeleton, cell cycle, ECM-receptor interaction, protein digestion and absorption, and several signaling pathways, including the Hedgehog signaling pathway, Rap1 signaling pathway, and PI3K-Akt signaling pathway (Figure 4D, right panel).

### Regulatory Networks of Putative miRNA-mRNA Interaction

**FIGURE 5:**
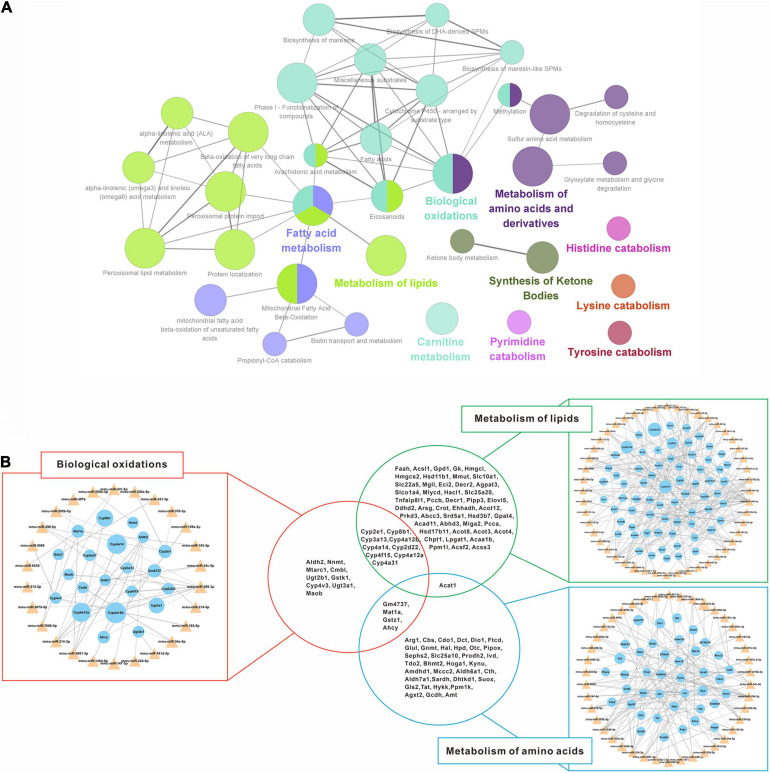
Regulatory networks of down-regulated miRNAs and up-regulated mRNAs interaction. **(A)** Enriched Reactome pathway interaction networks for up-regulated DEGs. Each node represented a Reactome pathway, and the colors indicated functional clusters of related biological pathways. The node size correlated with the enrichment significance, while the edge thickness reflected the strength of correlation between individual pathways. **(B)** Schematic overview of the miRNA-mRNA interaction networks in specific pathways. The triangles represented miRNAs and the circles represented the target mRNAs. The sizes of triangles and circles represented the fold changes in reversal mice compared to TAA mice, while the edge thickness represented Pearson correlation coefficient between miRNA and mRNA. DEGs, differentially expressed genes; TAA, thioacetamide.

To further reveal the regulatory role of DEMs and DEGs in the spontaneous reversal process of liver fibrosis, we constructed miRNA-mRNA interaction networks based on the Reactome pathway database. Indeed, most up-regulated DEGs were enriched in the metabolic pathways, mainly focusing on biological oxidations, lipid metabolism, and amino acid metabolism (Figure 5A). By integrating these biological pathways for both miRNA and mRNA categories (Figure 5B), we found that multiple miRNAs and their target mRNAs were involved in respective metabolic regulation, totally covering more than 90% of the identified DEMs (50/53). Remarkably, distinct pathways might interact with each other through the hub genes. Lipid metabolism and biological oxidations might interact with each other via Cytochrome P450 genes (*Cyp2e1*, *Cyp8b1*, *Cyp3a13*, etc.), while biological oxidations might interact with amino acid metabolism through *Gm4737*, *Mat1a*, *Gstz1*, and *Ahcy*. Unanimously, *Acat1* might serve as a linker between lipid metabolism and amino acid metabolism.

**FIGURE 6:**
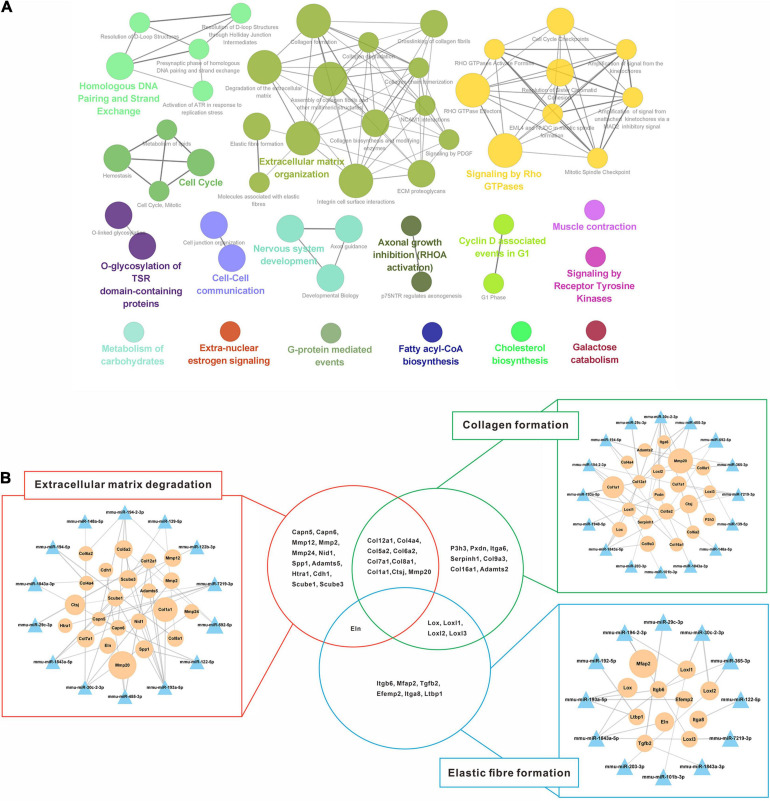
Regulatory networks of up-regulated miRNAs and down-regulated mRNAs interaction. **(A)** Enriched Reactome pathway interaction networks for down-regulated DEGs. Each node represented a Reactome pathway, and the colors indicated functional clusters of related biological pathways. The node size correlated with the enrichment significance, while the edge thickness reflected the strength of correlation between individual pathways. **(B)** Schematic overview of the miRNA-mRNA interaction networks in specific pathways. The triangles represented miRNAs and the circles represented the target mRNAs. The sizes of triangles and circles represented the fold changes in reversal mice compared to TAA mice, while the edge thickness represented Pearson correlation coefficient between miRNA and mRNA. DEGs, differentially expressed genes; TAA, thioacetamide.

Additionally, 1,056 down-regulated DEGs were mapped preferentially to pathways associated with the extracellular matrix, cell proliferation, biosynthesis, and kinase signaling pathway (Figure 6A). Since liver fibrosis reversal is a dynamic process involving coordinated changes in ECM synthesis and degradation, pathways related to ECM organization (collagen formation, elastic fiber formation and ECM degradation) were selected to validate the miRNA-mRNA regulatory networks (Figure 6B). 14 out of 23 DEMs were directly involved in ECM degradation, of which 12 DEMs regulated collagen formation, and 8 DEMs regulated elastic fiber formation. Additionally, the genes encoding collagen (*Col1a1*, *Col7a1*, *Col8a1, Col12a1*, etc.) and elastic fiber (*Eln*) were involved in the ECM degradation by regulation of collagen and elastic fiber formation.

### Key miRNA-mRNA Interaction Networks in Fibrosis Reversal

**FIGURE 7:**
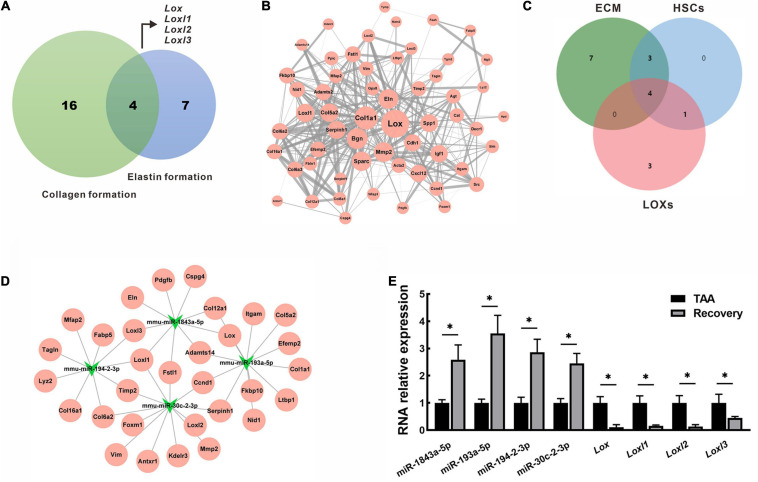
Identification and validation of key miRNAs and mRNAs in fibrosis reversal. **(A)** Venn diagram of DEGs between collagen formation and elastin formation to identify the hub genes. **(B)** PPI network of the hub genes (*Lox*, *Loxl1*, *Loxl2*, *Loxl3*) and their first-degree neighbors. The node size represented node degree in the PPI network, while the edge thickness reflected the combined scores between different nodes. **(C)** Venn diagram of DEMs targeting ECM component, HSCs activation and LOXs regulation. **(D)** miRNA-mRNA regulatory network of the overlapped miRNAs (mmu-miR-1843a-5p, mmu-miR-193a-5p, mmu-miR-194-2-3p, and mmu-miR-30c-2-3p) and their target genes. **(E)** Expression of miRNAs and mRNAs were validated by qRT-PCR in mice livers. Data were presented as mean ± SD. *n* = 6/group, *p < 0.05. DEGs, differentially expressed genes; DEMs, differentially expressed miRNAs; ECM, extracellular matrix; HSCs, hepatic stellate cells; LOXs, lysyl oxidases; Loxl1, lysyl oxidase-like 1; Loxl2, lysyl oxidase-like 2; Loxl3, lysyl oxidase-like 3; PPI, protein-protein interaction; qRT-PCR, quantitative real-time PCR; TAA, thioacetamide.

Collagen and elastin are major components of fibrotic tissues in the liver. Therefore, the DEGs intersections with collagen formation or elastin formation were assessed. 4 hub genes that belong to the lysyl oxidase (LOX) family (*Lox*, *Loxl1*, *Loxl2*, *Loxl3*, Figure 7A) were identified. Using the STRING database, PPI network analysis was performed for all down-regulated mRNAs, and a sub-network was further constructed by highlighting the first-degree neighbors of these key hub genes obtained from the PPI analysis (Figure 7B). Interestingly, we found that genes encoding ECM proteins and activated hepatic stellate cell (HSC) markers were closely clustered within the PPI network, suggesting their coordinate regulation on liver fibrosis reversal. To further clarify the miRNA-mRNA regulatory interactions, 4 overlapped miRNAs (mmu-miR-1843a-5p, mmu-miR-193a-5p, mmu-miR-194-2-3p, and mmu-miR-30c-2-3p) were identified based on a Venn diagram of DEMs targeting ECM component, HSCs activation and LOXs regulation (Figure 7C). These miRNAs and their target genes constructed a regulatory networks for miRNA-mRNA interactions (Figure 7D). A single miRNA might interact with different genes, while the same gene might be a target of different miRNAs. For example, mmu-miR-1843a-5p modulated *Lox*, *Loxl1*, and *Loxl3*, while *Loxl1* was the target of mmu-miR-1843a-5p, mmu-miR-194-2-3p, and mmu-miR-30c-2-3p. These data indicated complex interactions of miRNA-mRNA in the regulation of the spontaneous reversal of liver fibrosis.

### Validation of Key miRNA and mRNA

To validate the RNA sequencing results, we selected key miRNAs (mmu-miR-1843a-5p, mmu-miR-193a-5p, mmu-miR-194-2-3p, and mmu-miR-30c-2-3p) and mRNAs (*Lox*, *Loxl1*, *Loxl2*, and *Loxl3*)for qRT-PCR analysis in livers from the TAA group and the recovery group. As shown in Figure 7E, the RNA expression patterns of real-time PCR data were consistent with that of RNA sequencing data. Compared with TAA-fibrotic mice, miRNAs expression was significantly up-regulated while mRNAs expression was down-regulated in reversal mice, *p* < 0.05 (Figure 7E).

## Discussion

As an important pathological feature and an intermediate development stage for most chronic liver diseases, liver fibrosis develops and progresses due to the imbalance between ECM production and degradation (Karsdal et al., 2015). There is growing clinical evidence that early to moderate hepatic fibrosis can regress and possibly even resolve in a number of liver diseases based on histologic assessment (Ellis and Mann, 2012; Kisseleva and Brenner, 2021). In recent years, high-throughput sequencing technology enables a deep and efficient exploration at the molecular level for the regulatory mechanisms in fibrosis reversal (Stark et al., 2019). This study performed an integrative analysis of miRNA and mRNA expression profiles in the mouse model induced by TAA. A series of putative miRNAs and their target mRNAs were screened out. Their interactions and potential functions in the spontaneous reversal of liver fibrosis were revealed.

It has been known that miRNAs are epigenetic modulators that inhibit the expression of target genes. Although a lot of miRNAs have been identified in liver fibrosis progression, there is less report on miRNA-mRNA interactions and their regulatory mechanisms involved in fibrosis reversal. 102 differentially expressed miRNAs (DEMs), with 49 significantly up-regulated and 53 significantly down-regulated were identified in this study. Among them, miR-200a-5p and miR-122-3p have been reported as potential biomarkers associated with fibrosis progression (Van Keuren-Jensen et al., 2016). Interestingly, the miRNAs that regulate HSCs phenotype were consistently reversed with a concomitant decrease in hepatic ECM deposition. miRNAs that activate HSCs (miR-199a-3p and miR-214-3p) were significantly suppressed (Ma et al., 2018; Yang et al., 2020), while those restrain the proliferation and activation of HSCs (miR-378a-3p, miR-139-5p, miR-455-3p, and miR-193a-3p) were remarkably increased during the fibrosis regression (Hyun et al., 2016; Ju et al., 2019; Wei et al., 2019; He et al., 2021). Among the differentially expressed genes (DEGs), we obtained 2,845 differential genes between reversal mice and TAA-fibrotic mice. According to the integrative analysis, 3,769 putative negatively correlated miRNA-mRNA pairs containing 76 DEMs and 1,490 DEGs were revealed to be potentially implicated in the pathogenesis of this spontaneous reversal process. Nevertheless, 26 out of 102 DEMs failed to regulate the target genes, and only 52.4% of the DEGs were regulated by the corresponding miRNAs. These results suggested that a single miRNA could target multiple mRNAs, while multiple miRNAs could target a single mRNA. However, not all target genes had contrasting expression patterns compared to miRNA, which might be due to the synergistic or antagonistic regulatory effects of individual miRNAs with concordant or opposed function on the same intronic targets (Fabian et al., 2010).

GO enrichment and pathway analysis indicated that small-molecule metabolic pathways related to biological oxidation were strongly up-regulated during the reversal process of fibrosis. Substantial evidence has shown that lipotoxicity, originated from the imbalance between lipid uptake and utilization, is a key contributor to the development of fatty liver diseases by inducing endoplasmic reticulum stress, mitochondrial dysfunction and oxidative stress (Marra and vegliati-Baroni, 2018). As a canonical pathway involved in lipid metabolism, PPAR activation exerts potent anti-inflammatory, anti-steatosis, and anti-fibrosis effects due to its extended protection for hepatocytes, liver sinusoidal endothelial cells (LSECs), and HSCs via multi-pathways (Han et al., 2020). Several clinical trials of NASH indicated that PPAR agonists may improve steatosis, inflammation and fibrosis (Ratziu et al., 2016; Gawrieh et al., 2021). Our results provided valuable evidence that the PPAR pathway took part in the fibrosis regression regulation, and suggested that stimulation of PPAR pathway might serve as a potential therapeutic strategy for human liver fibrosis. Besides, the down-regulated DEGs were mainly enriched in signal transduction pathways related to ECM organization. Among these, Hedgehog and PI3K-Akt are two important stress-responsive pathways in the liver. During liver wound healing, both pathways are activated, ultimately leading to the transdifferentiation of HSCs into myofibroblasts, LSECs capillarization, and M2-polarization of macrophages (Yu et al., 2015; Machado and Diehl, 2018; Wang et al., 2019). Our results validated that deregulation of Hedgehog and PI3K-Akt signaling pathways was consistent with the ameliorative pathological characteristics of liver fibrogenesis in reversal mice. Although the small molecule inhibitors of Hedgehog and PI3K-Akt have been developed for use as anticancer agents, it is worthwhile to investigate their anti-fibrotic efficacy in future clinical trials.

Through the integration of the deregulated signaling pathways, we identified a series of genes in LOX family that affect collagen and elastin formation. Interestingly, all of these genes were significantly associated with those encoding ECM structures and activated HSCs markers in the PPI network. Supportively, LOXs-mediated cross-linking of both collagen and elastin throughout the initiation, progression, and regression of liver fibrosis (Chen W. et al., 2020). Over-expression of LOXs leads to an abnormal ECM synthesis and subsequent cross-linking, forming a vicious circle. The resultant ECM stabilization contributes to fibrosis progression and retards fibrolysis, for example, through matrix metalloproteinases-induced proteolytic degradation (Ikenaga et al., 2017; Zhao et al., 2018). As LOXs-specific miRNAs, mmu-miR-1843a-5p, mmu-miR-193a-5p, mmu-miR-194-2-3p, and mmu-miR-30c-2-3p identified in this study might be the potential pharmacological targets for the reversal of liver fibrosis.

In conclusion, the expression profiles of both miRNAs and mRNAs in the mouse model with the spontaneous reversal potency of liver fibrosis have been identified. Some differentially expressed miRNAs and their target mRNAs were correlated with fibrosis regression. A core functional miRNA-mRNA regulatory network which was meaningful to understand the molecular mechanisms for liver fibrosis reversal was constructed. The potential candidate targets for the treatment of liver fibrosis provided in this study need further elucidation.

## Data Availability Statement

The raw miRNA and mRNA sequencing data have been deposited in the NCBI Gene Expression Omnibus (GSE173961 and GSE173962).

## Ethics Statement

The animal study was reviewed and approved by the Animal Use and Care Committee of West China Hospital, Sichuan University.

## Author Contributions

CT and JG conceived and supervised the study. YT, CZ, TL, LZ, and YX performed the experiments. YT, HT, and RL analyzed the data. YT, JG, and CT wrote the manuscript with input from all the authors. All authors contributed to the article and approved the submitted version.

## Conflict of Interest

The authors declare that the research was conducted in the absence of any commercial or financial relationships that could be construed as a potential conflict of interest.

## Footnotes

1 http://mirwalk.umm.uni-heidelberg.de/

2 https://metascape.org/

3 https://cloud.oebiotech.cn and http://www.bioinformatics.com.cn

4 https://string-db.org/

## Supplementary Material

Supplementary Figure 1 Quality control and quantification of RNA samples.

Supplementary Table 1 Primer sequences for qRT-PCR.

Supplementary Table 2 Differentially expressed miRNAs (DEMs) in fibrosis reversal.

Supplementary Table 3 Differentially expressed genes (DEGs) in fibrosis reversal.

Supplementary Table 4 Putative negative miRNA-mRNA pairs by integrated analysis.

Supplementary Table 5 GO enrichment analysis of differentially expressed genes (DEGs) in fibrosis reversal.

Supplementary Table 6 KEGG pathway analysis of differentially expressed genes (DEGs) in fibrosis reversal.
