# Supplementary material for: Integrated Analysis of Hepatic miRNA and mRNA Expression Profiles in the Spontaneous Reversal Process of Liver Fibrosis
Source: Front Genet. 2021 Jul 22;12:706341. doi: 10.3389/fgene.2021.706341 (PMC8340883; doi:10.3389/fgene.2021.706341)
Supplement: Supplementary Figure 1 — Quality control and quantification of RNA samples. [file Data_Sheet_1.ZIP › Supplementary material/Supplementary Figures.docx]

**Supplementary Figures**

**
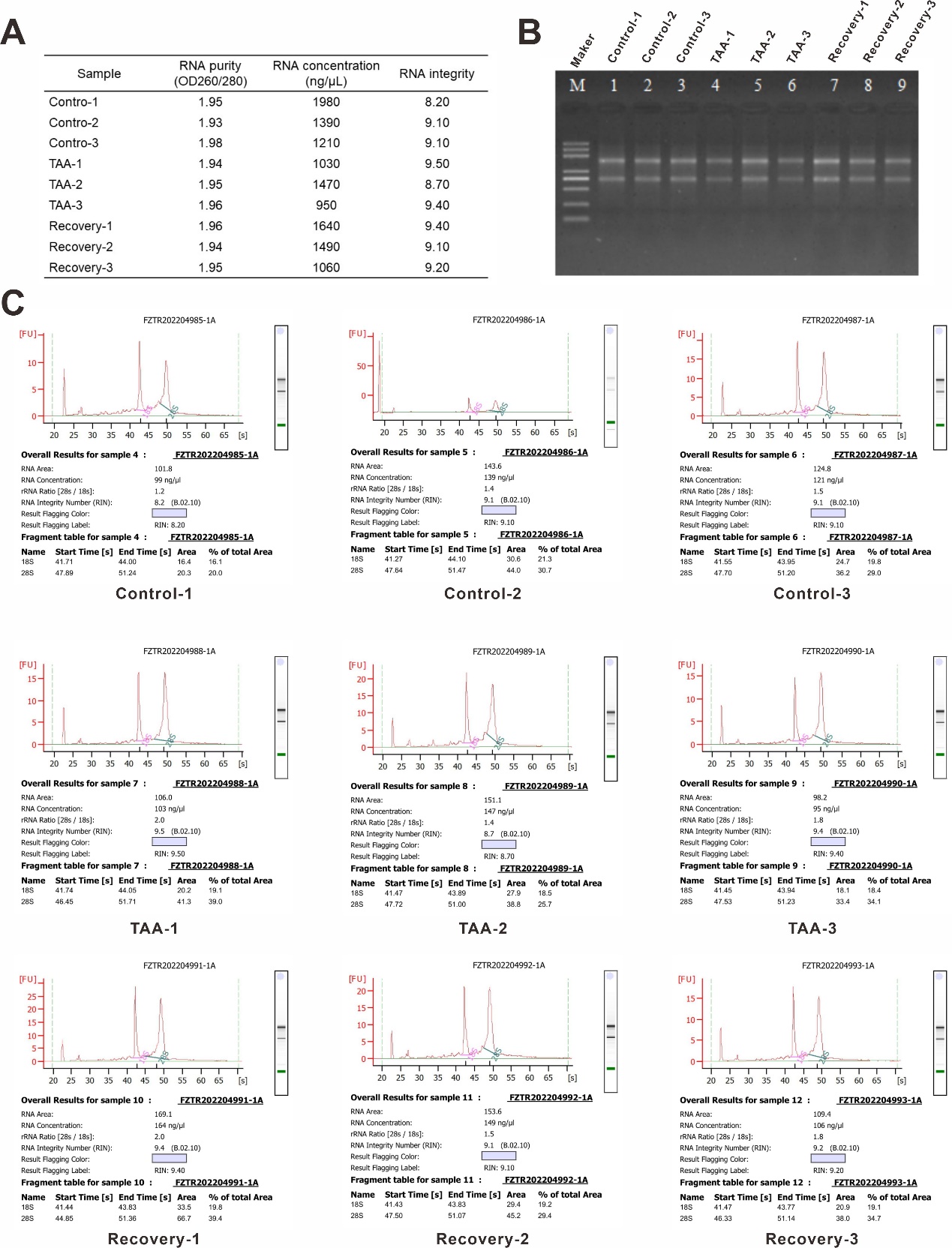
**

**Figure S1. Quality control and quantification of RNA samples.**

A: Detailed data proving the quality of RNA (purity, concentration and integrity). B: RNA degradation and contamination were monitored on 1% agarose gels. C: RNA integrity and quantity were determined by Agilent 2100.
