# Supplementary material for: Integrated Analysis of Hepatic miRNA and mRNA Expression Profiles in the Spontaneous Reversal Process of Liver Fibrosis
Source: Front Genet. 2021 Jul 22;12:706341. doi: 10.3389/fgene.2021.706341 (PMC8340883; doi:10.3389/fgene.2021.706341)
Supplement: Supplementary Figure 1 — Quality control and quantification of RNA samples. [file Data_Sheet_1.ZIP › Supplementary material/Table S1.docx]

**Table S1. Primer sequences for qRT-PCR**

| **RNA symbol** | **NCBI Reference Sequence** | **Sequence (5’-3’)** | | **Expected Product**  **Size** |
| --- | --- | --- | --- | --- |
| mmu-miR-1843a-5p |  | F | TATGGAGGTCTCTGTCTGACT | 21 bp |
| mmu-miR-193a-5p |  | F | TGGGTCTTTGCGGGCAAGATGA | 22 bp |
| mmu-miR-194-2-3p |  | F | CCAGTGGGGCTGCTGTTATCTG | 22 bp |
| mmu-miR-30c-2-3p |  | F | CTGGGAGAAGGCTGTTTACTCT | 22 bp |
| U6 snRNA | NC_000083.7 | F | TCGCTTCGGCAGCACATA | 59 bp |
|  |  | R | GGGGCCATGCTAATCTTCTC |  |
| *Lox* | NM_010728.3 | F | ACACGTCCTGTGACTATGGG | 168 bp |
|  |  | R | TGGGGTTTACACTGACCTTTA |  |
| *Loxl1* | NM_010729.3 | F | CCCATCTGTACTCCTTGCG | 223 bp |
|  |  | R | TCGTAGTGGCTGAACTCGTC |  |
| *Loxl2* | NM_033325.2 | F | GCTGTTTGGCTCTGCTTGT | 187 bp |
|  |  | R | CCCTCATTGTGCTTCCTCTT |  |
| *Loxl3* | NM_013586.5 | F | GCCACGTAGTCTGCGGTAT | 250 bp |
|  |  | R | TGACCAGTAAAGGTGGCATAA |  |
| *Gapdh* | NM_001289726.1 | F | CCTCGTCCCGTAGACAAAA | 252 bp |
|  |  | R | TCGCTCCTGGAAGATGGT |  |

qRT-PCR, quantitative real-time PCR; F: Forward; R: Reverse; Lox, lysyl oxidase; Loxl1, lysyl oxidase-like 1; Loxl2, lysyl oxidase-like 2; Loxl3, lysyl oxidase-like 3; Gapdh, glyceraldehyde-3-phosphate dehydrogenase.
